# Psoriasis

**Published:** 1879-06

**Authors:** N. E. Wordin

**Affiliations:** Bridgeport, Conn.


### PSORIASIS.

By N. E. WORDIN, M.D., Bridgeport, Conn.

All physicians who have had experience with this disease know the difficulty with which it yields to treatment. As I was very fortunate in the case to which I now refer, I take pleasure in recording it, although so simple. The patient was a lawyer of this city, in comfortable circumstances, and living well ; in age about fifty, spare in build, but healthy and capable of much endurance. He is a sufferer from malaria, but aside from that is in good health. Some months ago he showed to me a spot of psoriasis on the left temple. It was characteristic, and in size about as large as a half dime. I undertook to cure it, not fully realizing what trouble the little thing would give me. A favorite prescription with me in most all skin eruption is:

℞—Hydrarg. chlor. mit. - - - - - ʒj

Ung. aquæ rosæ - - - - - - ℥j

Mix.

This I directed to be frequently applied. For the calomel I afterwards substituted the same amount of white precipitate or hydrargrum ammoniatum. This he carried in his vest pocket and continually applied during the day. He was very faithful as he was very desirous of becoming rid of his annoyance. Having tried this satisfactorily without much result, I had resort to the formula of McCall Anderson, as found in No. 84, p. 512 of the edition of Tilbury Fox, 1873.

℞—Alcoholis - - - - - - -

Ol. cadini - - - - - -

Sap. mollis - - - - - - - aa ℥j

Ol. lavand - - - - - - ʒ ss

Mix.

This soon showed the stimulating effect of the tar. It diminished the cell proliferation, but left the red hyperæmic of the skin.

The scales had now all disappeared, and in the centre of the red areola was a smooth horny accumulation a little larger than the lead in an ordinary pencil. It occurred to me that if the enlarged papillæ and hypertrophied skin and vessels could be distroyed, the entire part might be built up *de novo* and the disease thus cured. I accordingly touched the centre before discribed with nitric acid. I repeated this application every time a new scale formed, and in the meantime the mercury ointment was applied. When last seen the spot of redness could scarcely be detected, and the slightest roughness in its centre was the only indication of anything abnormal. The treatment was thus almost entirely local. Arsenic was administered in the form of Fowler's solution, which improved him very much, but it was not continued long.—*N. Y. Med. and Surg. Brief.*

